# Stem cell therapies for autoimmune hepatitis

**DOI:** 10.1186/s13287-021-02464-w

**Published:** 2021-07-07

**Authors:** Ahmed Lotfy, Aya Elgamal, Anna Burdzinska, Ayman A. Swelum, Reham Soliman, Ayman A. Hassan, Gamal Shiha

**Affiliations:** 1 Biotechnology and Life Sciences Department, Faculty of Postgraduate Studies for Advanced Sciences (PSAS), Beni-Suef University, Beni-Suef, 62511 Egypt; 2 Department of Animal Histology and Anatomy, Faculty of Veterinary Medicine, Badr University in Cairo (BUC), Cairo, Egypt; 3 Department of Immunology, Transplantology and Internal Diseases, Medical University of Warsaw, Nowogrodzka 59, 02-006 Warsaw, Poland; 4 Department of Theriogenology, Faculty of Veterinary Medicine, Zagazig University, Zagazig, 44511 Egypt; 5 Department of Animal Production, College of Food and Agriculture Sciences, King Saud University, Riyadh, 11451 Saudi Arabia; 6 Tropical Medicine Department, Faculty of Medicine, Port Said University, Port Said, Egypt; 7 Egyptian Liver Research Institute and Hospital (ELRIAH), Mansoura, Egypt; 8 Hepatology and Gastroenterology Unit, Internal Medicine Department, Faculty of Medicine, Mansoura University, Mansoura, Egypt

**Keywords:** Stem cells, Autoimmune hepatitis, Mesenchymal stem cell, Hematopoietic stem cells, Exosomes, Mesenchymal stromal cell

## Abstract

Autoimmune hepatitis is a chronic inflammatory hepatic disorder which may cause liver fibrosis. Appropriate treatment of autoimmune hepatitis is therefore important. Adult stem cells have been investigated as therapies for a variety of disorders in latest years. Hematopoietic stem cells (HSCs) were the first known adult stem cells (ASCs) and can give rise to all of the cell types in the blood and immune system. Originally, HSC transplantation was served as a therapy for hematological malignancies, but more recently researchers have found the treatment to have positive effects in autoimmune diseases such as multiple sclerosis. Mesenchymal stem cells (MSCs) are ASCs which can be extracted from different tissues, such as bone marrow, adipose tissue, umbilical cord, and dental pulp. MSCs interact with several immune response pathways either by direct cell-to-cell interactions or by the secretion of soluble factors. These characteristics make MSCs potentially valuable as a therapy for autoimmune diseases. Both ASC and ASC-derived exosomes have been investigated as a therapy for autoimmune hepatitis. This review aims to summarize studies focused on the effects of ASCs and their products on autoimmune hepatitis.

## Introduction

Liver is one of the main effector sites of numerous systemic immune reactions that can lead to variable chronic and autoimmune disorders. Examples of autoimmune liver diseases (AILDs) are autoimmune hepatitis (AIH), IgG4-related sclerosing cholangitis, primary biliary cholangitis, and primary sclerosing cholangitis. These diseases can lead to hepatic dysfunctions including cholestasis, fibrosis, cirrhosis, and even liver cancer [1].

For chronic hepatic disorder, stem cell therapy seems to be a safe and effective treatment choice [2, 3]. Mesenchymal stem cells (MSCs) therapy possess numerous advantageous properties such as multipotential for differentiation, anti-fibrosis properties, and immunomodulatory effects [4]. Numerous investigations have shown that MSC therapy in AILDs is both safe and effective. Hematopoietic stem cells (HSCs) which are another type of stem cells have also a therapeutic capability. HSCs transplantation have been applied in several autoimmune diseases [5].

Many previous reviews are focused on the stem cell as a treatment for chronic hepatic disorders or acute-on-chronic liver failure [2, 6]. The current review aimed to summarize studies focused on the effects of ASCs and their products on autoimmune hepatitis.

## Autoimmune hepatitis

Autoimmune hepatitis (AIH) is one of the chronic hepatic disorders believed to be induced by a loss of tolerance to autoantigens specific to hepatocytes, and characterized by elevations of the levels of alanine aminotransferase (ALT), aspartate aminotransferase (AST), and immunoglobulins, particularly IgG, in the serum. The latter is used to determine biochemical remission [7]. Autoantibodies, including antinuclear antibodies (ANAs), smooth muscle antibodies, anti-liver kidney microsomal type 1 (anti-LKM1), and anti-cytochrome P4502D6 antibodies can be identified in the serum of people suffering from AIH [8].

Hepatic injury caused by inflammation [9], as well as various non-AIH-specific features such as lymphoplasmacytic infiltrations, hepatocyte resetting, and emperipolesis, are all important characteristics of AIH [10]

Besides plasma cells producing autoantibodies, the cytotoxic T cells specific to hepatocyte-expressed antigens were demonstrated to play an important role in the accelerating the hepatic damage during the course of AIH. The proposed cellular mechanisms behind the breaking of immune tolerance in AIH include the increased number of Th17 cells and defects in T regulatory cells activity.

Although the specific cause of AIH is unidentified, studies throughout the latter four decades have shown that the etiology of the diseases in both adult and juvenile is influenced by interconnections among genetic and environmental factors. Molecular mimicry, immunological activation in response to self-antigen presentation, and loss of self-tolerance are examples of these interactions as described in detail by Mieli-Vergani et al. [11].

Although the use of corticosteroids is the common treatment for AIH, some patients with disease react badly to such treatment, and some recovered patients may face severe adverse effects or recurrence following discontinuing steroid use [12]. For these reasons, novel therapies are needed.

**Fig. 1:**
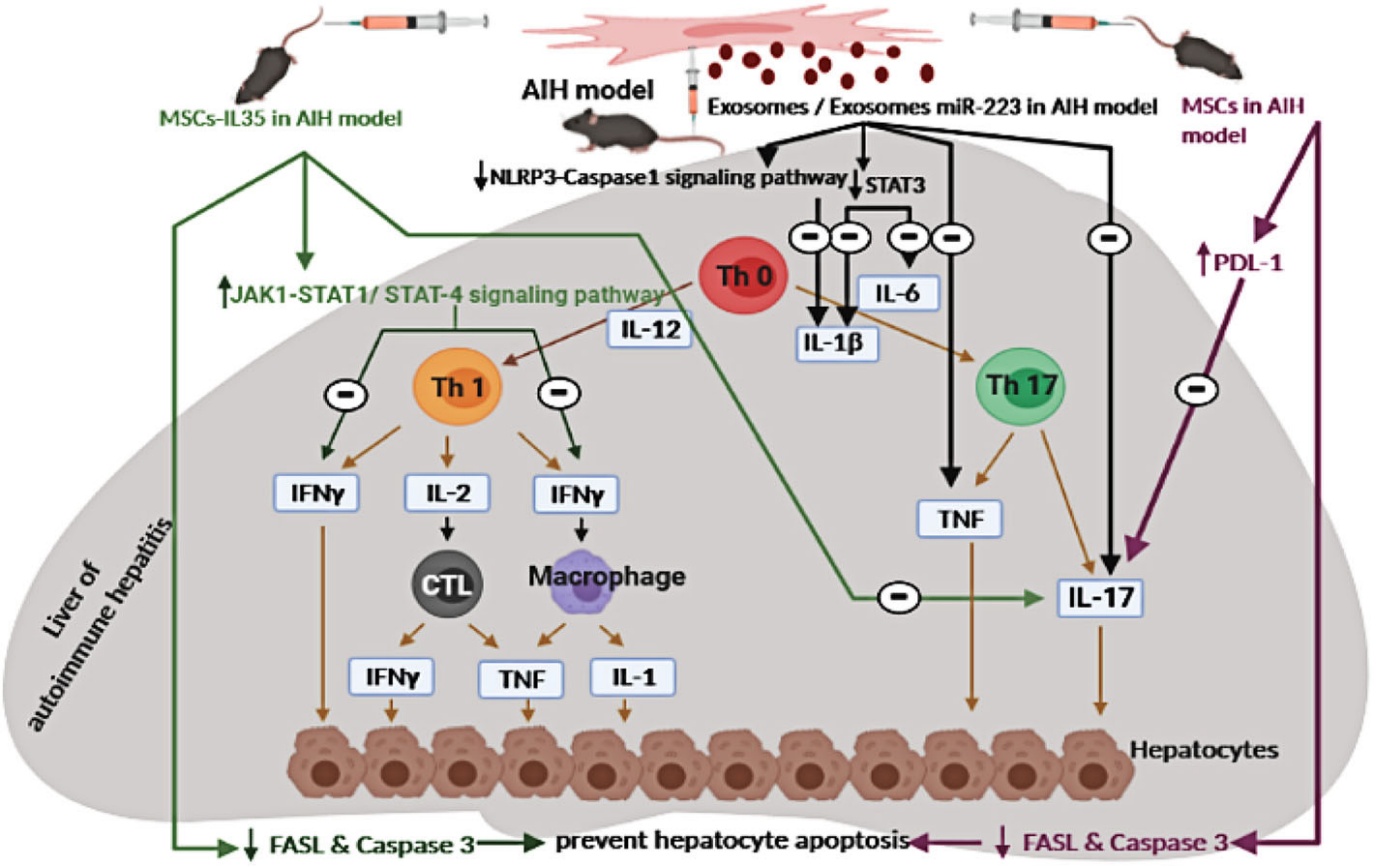
Postulated immunomodulatory role of MSCs, modified MSCs, and their exosomes in an AIH animal model. AIH, autoimmune hepatitis; Th17, T helper 17 cells; IL, interleukin; CTL, cytotoxic CD8+ T lymphocyte; JAK, Janus kinase; STAT, signal transducer and activator of transcription; FASL, Fas ligand; IFN-γ, interferon-gamma; PD-L1, the ligand of PD-1 “programmed death-1”; TNF, tumor necrosis factor; NLRP3, nucleotide-binding domain-like receptor protein 3. The figure was created using the images from Servier Medical Art

There are several AIH animal models produced to enable studying the hepatic immunity and pathological pathways of the disease (Fig. 1). AIH models include spontaneous models, models induced by surrogate antigens (including hepatitis induced by concanavalin A and α-galactosylceramide), and models induced by liver autoantigens (including hepatitis induced by liver homogenate, liver autoantigen, and mouse hepatic virus A59 infection) [13].

Concanavalin A (ConA) activates antigen non-specific T lymphocytes and macrophages resulting in acute immune-mediated liver damage [14]. Hepatic aggregation of Con A and its binding to mannose-rich glycoproteins on liver sinusoidal endothelial cells (LSECs) and Kupffer cells causes T cells to go into a cellular arrest. T cells, particularly NKT cells, produce inflammatory cytokines (e.g., IFN-γ and TNF-α), which activate other cells and cause apoptosis in LSECs and hepatocytes [15]. TNF receptors on hepatocytes may be upregulated by IFN-γ, resulting in fragmentation of the DNA and liver toxicity [16].

The use of liver extracts can cause pathological symptoms of AIH. As mouse model, a monthly injection of syngeneic liver homogenate with *Klebsiella pneumoniae* polysaccharide as adjuvant causes infiltration of primarily lymphocytes, in portal regions and piecemeal necrosis [17]. A different model is developed by intraperitoneal injection of mice with syngeneic liver homogenate (S-100) with Freund’s adjuvant. This results in inflammatory infiltrates and liver cell necrosis as well as production of S-100 protein-specific T cells [18].

## Stem cells

Stem cells are undifferentiated cells which have the ability to self-renew and to differentiate into specialized cell types [19]. Stem cells are classified based on their potency, into totipotent, pluripotent, multipotent, and unipotent stem cells. Stem cells are moreover divided into groups based on their origin into embryonic, fetal, umbilical cord (infant), adult, and induced pluripotent stem cells [20].

Stem cells are needed for the maintenance of homeostasis in tissues. They are usually in a quiescent state, and preserve their progenitor properties by self-renewing. Stimulation with extrinsic factors or damaged host cells activates stem cells to proliferate and differentiate, to renew the injured tissue. Stem cells are now also believed to actively interact with the tissue microenvironment, especially with components of the immune system [19].

### Mesenchymal stem cells

Mesenchymal stem cells (MSCs) are attracting a lot of attention because of their relative ease of isolation, extensive capacity for expansion, and capability of differentiation into multiple types of cell principally osteoblasts, chondrocytes, and adipocytes [21, 22]. MSCs have the potential to modify tissue regeneration and influence different immune problems, due to their immunoregulatory activity. MSCs have been reported to affect the behavior of different immune cells. In regards to the potential treatment of autoimmune diseases, including AIH, the most important features of MSCs seem to be the capability to suppress the activation and proliferation of lymphocytes and the promotion of Tregs formation. These abilities have been related to the expression/secretion of several molecules by MSCs: *CTLA*-*4* (cytotoxic T cell antigen 4), PD-L1 (ligand for programmed cell death protein 1), IDO-1 (indoleamine 2,3-dioxygenase 1), FasL (Fas ligand) iNOS (inducible nitric oxide synthase), TGF-β (transforming growth factor beta), and PGE2 (prostaglandin E2).

The inhibitory receptor — CTLA4 — was shown to be expressed by human MSCs both in cell surface and secretory form [23]. It is known that the inhibition of lymphocytes activation via CTLA4 is an important mechanism to maintain immune self-tolerance. It was suggested that mutations in *CTLA4* may contribute to the autoimmune hepatitis-like condition [24]. Human MSCs additionally express PD-L1 which is a ligand for PD-1 — an inhibitory receptor expressed on lymphocytes. Similarly to CTLA-4, MSCs express PD-L1 in both cell surface and secreted form [25]. The next factor which is believed to be responsible for suppressive effect of MSCs on lymphocytes is IDO-1, the enzyme converting tryptophan to kynurenine. Tryptophan is necessary for proper T cells function. IDO antagonist used in vitro diminished significantly suppressive effect of hMSCs on lymphocytes [26]. Additionally, it was postulated that IDO was one of key molecules responsible for therapeutic effect of MSCs administrated in colitis in mice model [27]. MSCs additionally express iNOS and FasL which also take part in their suppressive effect on lymphocytes. Inducible NOS generate nitric oxide (NO) which possess well known inhibitory impact on T cells by suppressing IL-2 pathways and inducing T cell apoptosis [28]. The Fas-FasL interaction is another axis crucial for controlling the lymphocytes number. The dysfunction of this mechanism leads to the autoimmune lymphoproliferative syndrome. It was shown that FasL expression mediates the suppressive effect of MSCs on the growth of multiply myeloma (plasma cells) in a mice model [29].

The expression of all these factors by MSCs results in their remarkable capability to suppress the activation and proliferation of lymphocytes which is a strong basis for their use in therapy of autoimmune diseases. In addition, while MSCs generally inhibit lymphocyte activity, they have been shown to promote a tolerogenic Treg population [30]. T regulatory cells are essential in maintaining peripheral self-tolerance. The effect of MSCs on Tregs differentiation can be either direct [31] or via switching monocytes toward anti-inflammatory macrophages [32]. It is believed that the process is mediated mainly through the secretion of TGFβ and PGE2, factors produced by MSCs in significant amounts. Moreover, PGE2 was also shown to take part in inhibiting the formation of Th17 subset under the influence of MSCs [33] which further support the tolerogenic influence of this population.

It is worth to underline that the expression of above described molecules by MSCs is significantly increased in pro-inflammatory environment. In vitro, it is stimulated by the addition of cytokines like IFN-γ and TNF-α and/or poly(I:C) — a ligand of TLR3 (toll-like receptor 3). This feature seems to be very promising in terms of potential therapeutic use in autoimmune diseases — MSCs would turn on their immunosuppressive activity in inflamed tissues.

MSCs are also able to interact with other immune cells, such as macrophages, natural killer cells, dendritic cells (DCs), neutrophils, and mast cells. All together, these interactions can promote tolerogenic activity of the immune system, thus facilitating the beneficial effect in various autoimmune disorders, as previously described [34–37]. Multiple studies have shown the remarkable immunomodulatory capacity of MSCs, and the ability of infused MSCs to suppress excessive inflammatory process and to promote tissue regeneration in various models of disorders such as graft-versus-host disease (GvHD) [38], rheumatoid arthritis [37], and multiple sclerosis [39]. However, the immunological status of the local environment and presence or absence of certain stimuli affects the ability MSCs to modulate the inflammatory process [19]. Unfortunately, there are also reports indicating that MSCs may enhance the growth of some tumors [40]. These data are in agreement with MSC tolerogenic and proangiogenic activity. Therefore, for upcoming clinical trials, short- and long-term side effects must be carefully observed and timely addressed.

#### MSC gene modifications

Owing to the features described in Section 3.1, MSC therapies have attracted considerable attention for several decades from researchers interested in the treatment of various diseases [41]. The functionality of MSCs can be increased by transforming them with genes that enhance their therapeutic potential. Previous studies have shown that MSC gene modifications, including the integration of exogenous genes such as hepatocyte growth factor [42], IL-7 [43], IL-10 [44], and Foxp3 [45] successfully improve the therapeutic performance of MSCs. MSCs with the IL-35 gene integrated into the genome (IL-35-MSCs) can successfully suppress the proliferation and functionality of CD4+ T cells in vitro [50].

**Table 1:** Role of MSCs, MSC gene modifications, and exosomes in experimental autoimmune hepatitis studies

	Disease modeling	Groups	MSCs				Sacrifice	Animal strain	Efficacy outcome	Mechanism	Ref.
			SC source	Number of cells/dose of exosome	Route of inj	Time of inj of stem/exosome					
**MSC**	IP injection of the S100/adjuvant for EAH model induction.	1, Control group	BM-MSCs		**IV**		On day 42	C57BL/6 mice	BMSCs could reduce EAH in a dose-dependent way. The therapeutic efficacy of MSCs given three times was superior to that of MSCs provided once.	MSCs stimulate PD-L1 and by turn inhibit the pro-inflammatory interleukin 17.	[46]
		2. Model group									
		3. Drug-treated group (prednisolone and azathioprine)									
		4. Once MSC-treated group		1 × 10^5^		On day 21					
		5. Double MSC-treated group		1 × 10^5^		On days 21 and 28					
		6. Triple MSC-treated group		1 × 10^5^		On days 21, 28, and 35					
**MSC gene modifications**	IV injection with Con A (15 mg/Kg body weight)	1, IL-35-MSCs 2, MSCs 3, PBS	AD-MSC		IV	Stem cells injected 2 h before Con A inj		C57BL/6J mice	Both IL-35-MSCs and MSCs have a protective effect in the Con A-induced fulminant hepatitis, but IL-35-MSCs exerted stronger therapeutic effects than MSCs.	- MSCs could alleviate the hepatic injury by reducing the IL-17 secretion of liver MNCs, but not IFN-γ. - IL-35-MSCs could exert stronger protection through regulating the secretion of both IL-17 and IFN-γ, which might be the results of combined action. - IL-35-MSCs prevented the hepatocytes apoptosis by decreasing the FasL expression by MNC and decreased the IFN-γ expression level through the JAK1-STAT1/STAT4 signal pathway.	[47]
**Exosome**	IP injection of the S100/adjuvant for EAH model induction	1. Control group 2. Model group 3. BMSC-exo-treated group 4. BMSC-exo^miR-223(+)^-treated group 5. BMSC-exo^miR-223(-)^	BM-MSCs exosomes	**-**	**-**	On days 21, 28, and 35	On day 21, 28, and 35	C57BL/6 mice	In mice and hepatocytes, both groups 3 and 4 effectively reversed S100 or LPS/ATP-induced damage meaning that BMSC-derived exosomes can protect the liver in EAH.	The exosomal miR-223 suppressed the NLRP3 activation by binding to its 3′-UTR, resulting in NLRP3 mRNA degradation hence a reduction in liver inflammation and cell death.	[48]
	**AML12** (mouse hepatocytes) is a cell line derived from hepatocytes from a male mouse.	**LPS/ATP-treated AML12 cells** were incubated with the following: 1. Control medium 2. BMSC-exo (20 μg/ml) 3. BMSC-exo^miR-223(+)^ (20 μg/ml) 4. BMSC-exomiR^-223(-)^ (20 μg/ml)									
	- In vivo A model of EAH was established by IP injection of the S100/adjuvant. **- In vitro** Macrophage RAW264.7 cells	1. Model 2. Prednisolone and azathioprin 3. MSC-exosomes 4. MSC-exosomes^miR-223-3p^ 5. MSC-exosomes^miR-223-3p(i)^	**BM-MSCs exosome**	2 μg/g body weight in 200 μl of PBS per animal	**IV**	On days 21 and 35	On day 42	C57BL/6 mice	Liver of EAH as well as macrophage show reduction in cytokine production in response to exosomes or exosomes^miR-223-3p^, beside reduction in hepatic inflammation of EAH	miR-223-3p downregulate the expression of the inflammatory gene STAT3 which is considered to be a major upstream activator of interleukin 1β and 6. Also, miR-223-3p causes elevation of the Treg/Th17 ratio. Furthermore, in macrophages, miR-223-3p inhibits the production of IL-6 and IL-1 produced by LPS.	[49]

*EAH* experimental autoimmune hepatitis, *inj* injection, *IV* intravenous, *IP* intraperitoneal, *BM-MSCs* bone marrow mesenchymal stem cells, *PD-L1* the ligand of PD-1 “Programmed death-1”, *PBS* phosphate buffer saline, *AD-MSCs* adipose-derived mesenchymal stem cells, *MNC* mononuclear cells, *Con A* concanavalin A, *FASL* Fas ligand, *IFN-γ* interferon-gamma, *IL* interleukin, *JAK* Janus kinase, *STAT* signal transducer and activator of transcription), LPS (Lipopolsaccharide), 3′-UTR (untranslated region ), MSC-exosomes^miR-223-3p(i)^ (MSC-exosomes with miR-223-3p knockdown), Treg (Regulatory T cells), Th17(T helper 17 cells).

#### Exosomes

Exosomes are described as lipid bilayer-encased membrane vesicles less than 150 nm. Almost all types of cells secrete them, and they can transmit bioactive substances such as proteins, mRNAs, and microRNAs, from one cell to another [51, 52]. MSC-exosomes could be used to reduce the adverse effects caused by the administration of MSCs, such as tumorigenesis, toxicity, undesired immune responses, and formation of emboli [53, 54]. Exosomes have been shown to modulate inflammation in many studies [55]. Exosomes are important effectors of the paracrine activity of BMSCs, and exosomes produced from BMSCs have been found to enhance recovery in animal models of GvHD and drug-induced liver injury according to recent research [56, 57]. In experimental animal models of autoimmune uveoretinitis, MSC-exosomes suppressed Th17 differentiation and lowered IL-17 secretion [58]. The functionality of exosomes can be expanded to the areas of immunology, neurobiology, stem cell biology, and tumor biology [59].

### Hematopoietic stem cells

Hematopoietic stem cells (HSCs) are multipotent stem cells that can differentiate into all blood cell types [60]. In human at adult stage, HSCs are found mainly in bone marrow, less in peripheral blood, and in umbilical cord blood during pregnancy. The proliferation of HSCs results in generation of both: precursors for all blood cell lineages and cells which maintain their undifferentiated state [61].

For serious and treatment-refractory autoimmune problems, hematopoietic stem cell transplantation (HSCT) has become as a viable therapy. The pathogenesis of autoimmune diseases is currently attributed to the incorrect identification of self-antigens by T and B cells causing cell-mediated or humoral reactions, or both, and resulting in inflammatory tissue and vascular damage [62]. HSCT is used to eliminate autoreactive immune cells and recreate a naive, self-tolerant immune system in patients with autoimmune disorders [63].

Multiple sclerosis, systemic sclerosis, and rheumatoid arthritis are among the autoimmune illnesses for which HSCT is a treatment option [64]. The American Society for Blood and Marrow Transplantation (ASBMT) stated that HSCT is approved as a “standard of care, clinical evidence available” for diseased people with treatment-refractory relapsing MS [65]. HSCT is indicated as a “standard of care” for individuals suffering from severe systemic sclerosis in the ASBMT position statement [66].

Anti-thymocyte globulin, along with either high dosage Cyc or other chemotherapeutic drugs, is the most common conditioning protocol applied in people with AIH indicated for autologous HSCT [64]. Following conditioning, autologous (CD34+) stem cells are infused. After their neutrophil count has been recovered, patients may be allowed to leave the hospital which typically occurs within one to three weeks following the stem cell administration. From many months following HSCT, majority of patients stay significantly lymphopenic, until their immune system is completely reconstituted [67].

In an autologous graft, stem cell mobilization, conditioning, and T-cell depletion is linked to a higher risk of infection [64]. Autoimmune disorder can develop de novo during immune recovery after HSCT, due to impaired functioning of mechanisms building immune self-tolerance. At 5 years following HSCT, the cumulative rate of recurrent autoimmune disease was observed to be 9.8% [68]. EBMT registry information has been analyzed and revealed that relapse of the disease following HSCT is an ongoing problem [69].

Despite the important role of stem cells in immune modulation, there are a few studies investigating the effects of stem cells on AIH, as summarized below.

## Stem cells and AIH studies

### Experimental autoimmune hepatitis (EAH) studies

#### MSCs and experimental autoimmune hepatitis

Chen et al. [46] tested the effects of different treatments in a mouse model of EAH. One group received three treatments of BMSCs, one group was treated twice with BMSCs, one was treated once with MSCs, and a group was treated with prednisolone and azathioprine. The model was established using intraperitoneal (IP) injection of the S100/adjuvant in C57BL/6 mice. Mice which underwent the induction of EAH with no subsequent treatment constituted control “model group”. The researchers found that administration of culture-expanded BMSCs can alleviate EAH in a dose-dependent way, and the therapeutic effect observed in the group that received an intravenous (IV) injection of 1 × 10^5^ BMSCs three times was better than single injection.

BMSC-treated groups showed a decrease in ALT and AST serum levels compared to the model group, and the treatment significantly alleviated the lymphocyte infiltration and the occurrence of intra-lobular inflammatory lesions and necrosis. The PD-L1 in EAH mouse livers was upregulated compared with the control group and was reversed in drug-treated and BMSC single-treatment groups. PD-L1 was considerably more elevated in the group that received MSCs in a triple manner than in the group that received MSCs only once. The increased PD-L1 expression corresponded with the reduced inflammation and tissue damage. The expression of interleukin 17 was elevated in EAH mice and gradually decreased over time, following MSC transplant. This trend was contrary to natural tendency of variation of PD-L1, revealing that MSCs stimulate PD-L1 that suppresses interleukin 17 production. In the model group, IL-17 levels were also increased, showing that it played a pro-inflammatory role in EAH mice and that pro-inflammatory and anti-inflammatory actions were present in EAH animals.

Chen et al. [46] concluded that BMSCs inhibited the immune response in their experiment by stimulating the inhibitory factor PD-L1 which subsequent inhibit the pro-inflammatory interleukin 17. PD-1 and PD-L1 negative co-stimulatory binding interferes with CD28-mediated PI3K activation which blocks the Akt phosphorylation, reducing T-cell multiplication and maintenance and glucose metabolism as well as cytokines synthesizing (Fig. 1) [70].

#### MSC gene modifications and experimental autoimmune hepatitis

Regulatory T (Treg) cells are a subset of CD4+ T lymphocytes that are necessary for keeping immune self-tolerance. IL-35 is a member of the IL-12 family of heterodimeric cytokines. It is generated by Treg cells and is needed for maximal suppressive activity in Tregs [71]. In contrast to the other IL-12 family members, IL-12 and IL-23, IL-35 appears to have a strictly regulatory function. The primary physiological role of IL-35 is to regulate T-cell homeostasis, by inhibiting the polarization of T helper (Th) 2 and Th17 cells [72]. IL-35 have anti-inflammatory role in autoimmune diseases such as EAE and CIA [73, 74]. However, the physiological impact of IL-35 on concanavalin A (Con A)-induced hepatitis was not known until Wang et al. [47] investigated the effect of IL-35 gene-programmed MSCs in preventing Con A-induced liver injury. Con A-induced hepatitis is a well-studied murine model that largely reflects human AIH disease [14].

Wang et al. [47] reported that both IL-35 gene-modified MSCs (IL-35-MSCs) and adipose-derived MSCs have a protecting effect in Con A-induced fulminant hepatitis in C57BL/6J mice. Hepatitis was induced by the IV injection of 15 mg/Kg body weight Con A 2 h after the administration of MSCs. IL-35-MSCs exerted therapeutic impact more powerful than adipose-derived MSCs (Fig. 1).

A survival study was carried out, in which the dosage of Con A was elevated to 20 mg/Kg body weight. In this experiment all of the IL-35-MSC-transplanted mice survived throughout the whole period of observation, while the PBS-treated animals died within 24 h. The survival rate of mice transplanted with MSCs amounted 40%. Administrated IL-35-MSCs were able to migrate to the damaged liver tissues in a more targeted manner than the MSC- and PBS-treated groups. In both IL-35-MSCs, which had the best treatment effect, and MSCs, the area of liver necrosis was reduced, the expression level of active caspase3 was reduced, and the Fas ligand (FasL) mRNA level of mononuclear cells (MNC) was decreased. The IFN-γ expression level in the hepatic MNCs and liver tissue showed a significant decrease in the IL-35-MSC-transplanted group, in comparison to the MSC and PBS groups. The IL-17 expression level in the hepatic MNCs and liver tissue was significantly decreased in both the IL-35-MSC- and MSC-treatment groups, and the differences between them were insignificant. There were no significant differences in the expression levels of TNF-α, IL-4, IL-2, and IL-12 in hepatic MNCs. The expression of the proteins JAK1, STAT1, and STAT4 was significantly increased in the IL-35-MSC-treated groups compared with the pure MSCs and the PBS control groups. The amount of protein expression was increased significantly after phosphorylation. There was no significant difference and no obvious detection of pJAK2 among the three groups.

Wang et al. [47] found that MSCs could alleviate liver injury by reducing the secretion of IL-17, but not IFN-γ, by liver MNCs, while IL-35-MSCs exerted stronger effects by regulating the secretion of both IL-17 and IFN-γ. IL-35-MSCs prevented the apoptosis of hepatocytes by reducing the expression of FasL by MNC and decreased the level of expression of IFN-γ via the JAK1-STAT1/STAT4 signal pathway (Fig. 1).

#### Exosomes and experimental autoimmune hepatitis

Two experiments have been conducted using MSC-derived exosomes as a therapy for EAH [48, 49]. In the first experiment Chen et al. [48] studied the effect of IV injection of BMSC-derived exosomes and exosomes isolated from BMSCs transfected with miR-223 (BMSC-exo^miR-223(+)^ ) in an EAH model established by IP injection of C57BL/6 mice with the S100/adjuvant and in LPS/ATP-treated AML12 cells. They reported that both BMSC-exo and BMSC-exo^miR-223(+)^ reversed both S100- and LPS/ATP-induced damage both in animals and in liver cells, indicating that BMSC-derived exosomes can protect liver injury in AIH animal model. Their result indicated that mice treated with either BMSC-exo or BMSC-exo^miR-223(+)^ exhibit increased amount of miR-223 in hepatic tissue and displayed significantly better liver structure. Additionally, Chen et al. reported a significant decrease in serum levels of ALT and AST (greater using BMSC-exo^miR-223(+)^), significant decreases in levels of the pro-inflammatory cytokines TNF-a, IL-17A, and IL-1β in serum, and mRNA levels in the liver with a more pronounced difference in BMSC-exo^miR-223(+)^ treated mice, and a significant reduction in protein and mRNA expression of NLRP3 and caspase-1 in the liver.

They also found that in vitro PKH67-labeled exosomes were up-taken and transported to the cytoplasm compartments, and miR-223 in AML12 cells increased when co-cultured with MSC-exo or MSC-exo^miR-223(+)^. Co-treatment with LPS and ATP substantially upregulated the expression of NLRP3, caspase-1, and IL-1β. The IL-1β level reduced in the MSC-exo group, with the MSC-exo^miR-223(+)^ group showing the greatest reduction. In comparison to the model group, the NLRP3 level and the caspase-1 level dropped. LPS and ATP caused severe damage to AML12 hepatocytes, while MSC-exo and MSC-exomiR^-223(+)^ saved cells from death caused by LPS and ATP.

The NLRP3-caspase-1 signaling pathway may be involved in the mechanism behind these findings. Exosomal miR-223 bound to NLRP3’s 3′-UTR and inhibits its activation, resulting in the degradation of NLRP3 mRNA and the suppression of liver inflammation and cell elimination. During the activation of NLRP3, inactive procaspase-1 is cleaved into p20 and p10 subunits, causing the maturation and production of IL-1β. As well as secreting pro-inflammatory cytokines, caspase-1 elicits a novel kind of inflammatory cell death, called pyroptosis [75]. Cell membrane rupture and the release of pro-inflammatory cellular contents are associated with pyroptosis, which is dependent on caspase-1 activation. In response to internal or external danger signals, it is thought to be a critical element in innate immune response, activating caspase-1 and producing interleukin IL-1β [76]. Many researchers have indicated that abnormal NLRP3 activation contributes to autoimmune disorders development [77, 78].

In the second experiment, Lu et al. [49] studied the effect of IV injection of BMSC-derived exosomes without modification and transfected with miR-223-3p (BMSC-exo ^miR-223-3p^) in an EAH model and in LPS-treated macrophage RAW264.7 cells. miR-223-3p (is an earlier name after the old synonyms miR-223 or the guide strand) was successfully incorporated into MSC-exosomes and delivered miR-223-3p into macrophages. The exosomes had no toxic effect on the macrophages and either the application of exosomes or exosomes with miR-223-3p improved the inflammatory responses in the EAH and lowered the release of inflammatory cytokines in both the liver and macrophages**.**

In response to injection of MSC-exosomes or MSC-exosomes^miR-223-3p^, dramatically there was improvement revealed by reduction in the infiltration of MNC into the centrilobular or portal areas, besides reduction in the levels of liver enzymes and inflammatory lesions. The results of serum analysis to the levels of some interleukins in response to the exosomes therapy show significant lowering in the levels of interleukin 1β, 6, and 17 and dramatic increasing in interleukin 10 in comparison to treatment with PBS or MSC-exsomes^miR-223-3p(i)^ (MSC-exosomes with miR-223-3p knockdown).

MSC-exosomes and MSC-exosomes^miR-223-3p^ were capable of reducing the proportions of Th17 cell while increasing Treg cell proportions. The Treg/Th17 ratio was significantly reduced in the EAH induced mice, but the ratio returned to normal after treatment with MSC-exosomes.

Macrophages engulfed MSC-exosomes that was tagged with GFP in vitro. At mRNA and protein levels there was reduction in the levels of interleukins 1β and 6 which was induced previously by LPS, after treatment with either MSC-exosomes or MSC-exosomes^miR-223-3p^. The authors hypothesized that the mechanism of improvement induced by BMSC-exo and BMSC-exo ^miR-223-3p^ could possibly being linked to miR-223-3p, which negatively modulates the expression of the inflammation-related gene STAT3, which has been identified as a key activator of interleukin 1β and 6.

Both IL-1β and IL-6 are involved in AIH. IL-6 has been shown to stimulate the differentiation of naïve T lymphocytes into Th17 cells [79, 80], which release IL-17 and aid in the progression of AIH [81]. Human Treg cells can be converted to Th17 cells by IL-1β [82]. T regulatory cells play a critical immune role via secretion of interleukin 10 which keep immune homeostasis and tolerance in the liver [83]. In experimental AIH, there was elevation in both T regulatory and T helper 17 cells and their secretions interleukin 10 and 17 while the ratio of T regulatory/T helper 17 was much lower [84–88]. STAT3 stimulates RORt and increases interleukin 17 release in T helper 17 cells, which has important role in autoimmune disease [58].

In summary, it has been shown that MSCs provide a therapeutic effect in AIH animal models. EAH had been alleviated by the administration of BMSCs in a dose-dependent way and this was explained that BMSCs had upregulated PD-L1 and inhibited IL-17. MSCs have been genetically modified with IL-35 to increase their efficacy by preventing the hepatocytes apoptosis by lowering the expression of FasL by mononuclear cells (MNC) and decreased IFN-ỵƔ expression level through the JAK1-STAT1/STAT4 signal pathway. Moreover, the BMSC-derived exosomes showed a promising therapeutic effect either containing additional miRs or none by inhibition of inflammatory cytokines (Fig. 1, Table 1).

### Case reports

#### Hematopoietic stem cell transplantation and AIH [89]

Calore et al. [89] reported that AIH could be cured with allogeneic HSCT (Table 2). This finding is particularly important for patients who have both AIH and a hematological illness at the same time. These researchers had a patient who was diagnosed with Hb SS sickle cell disease (SCD) as a 4-year-old girl, when she was hospitalized because of severe hemolytic anemia. At the age of 13 years, the patient was diagnosed with AIH type I, when hospitalized for cholestatic jaundice and elevation of liver enzymes. The diagnosis was done by liver histology, which revealed portal and peri-portal fibrosis, portal inflammatory infiltrates primarily composed of lymphocytes and plasma cells, and focal areas of necrosis. Steroids were limited and withdrawn 5 months after the AIH was diagnosed but had to be restarted 1 month later due to a relapse. Four years later, the patient had another two relapses of AIH, indicating that constant immunosuppression with steroids was needed. During the follow-up, the antinuclear and anti-smooth muscle autoantibodies were still detected as positive, with a varying titer related to disease activity. Exchange transfusions were conducted every 18–20 days due to the appearance of repeated and severe vaso-occlusive crises while being administrated steroids.

**Table 2:** Showing the role of HSCT and BMT in AIH

	Patient main disorder	Patient gender	Donor	Transplanted cells	Results	Ref.
**HSCT**	patient with SCD and AIH	Female	Patient’s HbSA haploidentical father	HSCT	- **After HSCT by 2 years**, the data showed a full donor hematopoiesis, no SCD manifestation was noted, cerebral vasculopathy appeared to be resolved and the patient required no additional medical treatment. Antinuclear and anti-smooth muscle autoantibodies were negative 2 months after HSCT, and there was no recurrence of AIH in the patient.	[89]
**BMT**	Patient with a 4-year history of AIH and developed acute AL-L2.	Male	His healthy 25-year-old sibling was chosen as an MLC-non-reactive BM donor who was HLA A-, HLA B-, and DR-matched	- 6·8 × 10^8^ bone marrow cells for each kilogram (overall 4·7 × 10^10^ cells) - 10^5^ T lymphocytes for each kilogram body from a donor's peripheral blood (overall of 69 × 10^5^ T lymphocytes)	- Allogeneic transplantation of BM and donor T cells resulted in the normalization of T-cell responses to ASGPR, the elimination of antibodies to the same autoantigen, and the noticeable treatment of AIH.	[90]

*AIH* autoimmune hepatitis, *SCD* sickle cell disease, *TCRαβ* T cell receptor, *PBSC* peripheral blood stem cell, *HSCT* hematopoietic stem cell transplantation, *GvHD* graft-versus-host disease, *AL-L2* acute lymphoblastic leukemia, *HLA* human leukocyte antigen, *MLC* mixed leukocytes culture, *BMT* bone marrow transplantation, *ASGPR* antiasialoglycoprotein receptor

After conditioning with a combination of treosulfan, fludarabine, and Graphalon® anti-thymocyte globulins, the patient had HSCT at the age of sixteen. The intensive transfusion program necessary to control the cerebrovascular disease, and the recurrent vaso-occlusive crises were the main indication for HSCT. No post-transplant immune suppression was used, and all AIH therapies were stopped before conditioning began. The patient’s HbSA haploidentical father was used as a donor, and an ex-vivo TCRαβ and CD19 depletion of PB stem cells was performed [91].

The patient developed a fever, stomach pain, and anorexia on day 35 after undergoing HSCT and was diagnosed with grade II acute GvHD. She was treated with cyclosporine and extracorporeal photochemotherapy, and within a week, the symptoms were completely gone. Two years after HSCT, chimerism analysis revealed full donor hematopoiesis, cerebral vasculopathy had nearly disappeared, and the patient had no medical treatment.

#### Bone marrow transplantation and AIH

Vento et al. [90] suggested that if standard immunosuppressive treatment fails, adoptive cellular immunotherapy may be a viable option for people with AIH. They presented a case report involving a 19-year-old man with 4 years history of AIH who tested negative for all viral hepatitis. In October 1991, he was diagnosed with acute lymphoblastic leukemia. His wholesome brother was chosen as an MLC-non-reactive BM donor who was HLA A-, HLA B-, and DR-matched. In January 1992, the patient got 6·8 × 10^8^ BM cells (which had been depleted of red blood cells) per kg (total 4·7 × 10^10^ cells) following conditioning with fractionated whole body irradiation, etoposide, cyclophosphamide, and fractionated total lymphoid irradiation. Anti-CDW52 rat antihuman lymphocyte antibody was used to remove T cells. As part of a strategy designed to increase the donor T lymphocyte fight against residual host leukemia cells.

There was no anti-GvHD prophylaxis provided after the BMT, but the patient suffered from grade 1 acute cutaneous GvHD following receiving the donor T cells and was medicated with prednisolone and cyclosporin A orally. From 1 month following BMT, asialoglycoprotein receptor specific suppressor-inducer T-cell activity has returned to normal on 27 occasions, and T-cell immunity to the same antigen has been undetected. In April 1993, histology of the liver revealed that it had improved (Knodell score 4), and in April 1996, liver histology indicated resolution of liver disease (score 1), and the leukemia did not return.

Although it appears that immunosuppressive therapy for BMT generated AH remission in this patient, the sustained remission, and the normalization of ASGPR-specific suppressor-inducer T-cell activity after BMT, supposed that the suppressor function that was missing in this patient was supplied by the donor T cells. It’s possible that clonal elimination of the liver-damaging helper T cells has been accomplished (Table 2) [90].

## Future perspectives

Few studies have been carried out into the effect of stem cells as therapy for AIH. More research is needed, with more efficient models, more standardized protocols, and longer duration, to exclude possible long-term side effects, before preclinical and clinical trials can be conducted.

MSC and EAH research has depended on the use of Con A-induced hepatitis and liver homogenate (S-100 emulsified in Freund’s adjuvant) as models for EAH. Con A-induced hepatitis is a useful model for highly specific activation of T cells and used widely in research for investigating the role of T cells in AIH, but it is acute hepatic injury model not chronic, also it is a cytokine-dependent and antigen-independent. The use of liver homogenate for inducing hepatitis is a simple approach and has been widely used in the search for the mechanisms of AIH, but these models are induced by transferring of liver extracts to the animal body with a mostly unidentified composition [92]. There is therefore a need for better EAH models. Methods of administration, number and source of stem cells, toxicity, immune responses, development of embolus, tumorigenesis, and other adverse impact of MSCs should be controlled before the application of MSCs in clinical studies.

MSC-exosome therapy appears to be promising, and the use of these bodies may be able to assist in lowering the risk of MSCs administration [49]. However, further investigation is needed to determine toxicity caused by MSC-exosome therapy, either organ-specific or systemic, and their long-term adverse effects and efficacy. Consideration should be given to how to more effectively and conveniently extract exosomes. Methods for delivering exosomes more specifically into target areas must be developed [48]. Different passages of MSCs, as well as the state of the cells, can influence the production and composition of exosomes. For example, Xin et al. [93] have shown that MSC-exosomes subjected to ischemic tissues, possess large amounts of miRNAs (miR-133b). The discovery of the molecular composition of exosomes will require more research.

In conclusion, MSC treatment has shown to be promising in various AIH animal researches. However, to yet, no clinical investigations relying on MSC treatment have been conducted. Therefore, the clinically beneficial effect of MSCs in therapy of AIH needs approval. Therefore, further basic and clinical studies focused on stem cell therapies for AIH are required. The possible synergistic effects between stem cells and other immunomodulation compounds have not yet been tested. Also, the use of MSCs to aid HSCs trasplantation and to avoid graft failure requires further testing.

## Data Availability

All data presented in this review are totally available and present in the text.

## Abbreviations

EAH: Experimental autoimmune hepatitis
inj: Injection
IV: Intravenous
IP: Intraperitoneal
BM-MSCs: Bone marrow mesenchymal stem cells
CTLA-4: Cytotoxic T cell antigen 4
IDO-1: Indoleamine 2,3-dioxygenase 1
PD-L1: The ligand of PD-1 “Programmed death-1”
PBS: Phosphate buffer saline
AD-MSCs: Adipose-derived mesenchymal stem cells
MNC: Mononuclear cells
Con A: Concanavalin A
FasL: Fas ligand
IFN-γ: Interferon-gamma
IL: Interleukin
iNOS: Inducible nitric oxide synthase
JAK: Janus kinase
PGE2: Prostaglandin E2
STAT: Signal transducer and activator of transcription
LPS: Lipopolsaccharide
3′-UTR: Untranslated region
Treg: Regulatory T cells
Th17: T helper 17 cells
TGF-β: Transforming growth factor beta
